# A Pilot Study Examining ADHD and Behavioural Disturbance in Female Mentally Disordered Offenders

**DOI:** 10.3934/publichealth.2014.2.100

**Published:** 2014-05-28

**Authors:** Jack Hollingdale, Emma Woodhouse, Philip Asherson, Gisli H. Gudjonsson, Susan Young

**Affiliations:** 1 King's College London, Institute of Psychiatry, De Crespigny Park, London SE5 8AF, UK; 2 Broadmoor Hospital, Crowthorne, West London Mental Health Trust, UK; 3 Centre for Mental Health, Imperial College London, London, UK

**Keywords:** Attention-deficit hyperactivity disorder (ADHD), critical incidents, females, offenders, forensic psychiatry, self-harm, aggression, prevalence, management

## Abstract

Compared with general population rates, prevalence rates of ADHD have been consistently reported to be higher in both male and female offender populations, the latter estimated to range between 10–29%. Research in forensic institutional settings has reported that aggressive behaviour is a particularly prominent source of impairment among men with ADHD. However there is a paucity of research investigating the type of behavioural incidents that may arise in female offenders with ADHD. This pilot study therefore aimed to further our understanding of ADHD within a cohort of female mentally disordered offenders by ascertaining estimated rates of ADHD and associated functional disturbance presenting in this population. Fifty female offenders completed the Barkley ADHD rating scales. Data on aggressive and self-harming behaviours were obtained from patients' clinical records. Almost one-third of patients (28%) screened positive for ADHD, most commonly hyperactive/impulsive and combined subtypes. They were significantly younger than their peers and there were no significant differences in behavioural disturbance records between groups. When controlling for age, hyperactive/impulsive symptoms and combined symptoms were significantly and positively correlated with measures of behavioural disturbance. ADHD symptoms correlated more strongly with self-harm than outward aggression, which is a novel finding. This pilot study has contributed to the knowledge base about the rate and functional problems of female offenders with ADHD. Future research should replicate the study using a larger sample and explore the effect of treatment (pharmacological and psychological) on the reduction of ADHD symptoms, behavioural disturbance, length of stay and quality of life.

## 1. Introduction

ADHD is a clinical syndrome defined in the DSM-5 [1] by impairing levels of hyperactive, impulsive and inattentive behaviours. The diagnosis can be classified into three subtypes of clinical presentation: predominantly inattentive, predominantly hyperactive/impulsive, and combined type. It is a common psychiatric disorder prevalent in around 2.5% of adults [2] and is associated with impaired functioning [3]–[4]. Compared with general population rates, prevalence rates of ADHD have been consistently reported to be higher in both male and female offender populations [5], the latter estimated to range between 10–29% [6]–[8].

Much of the extant literature has focused on male forensic populations, and in institutional settings aggressive behaviour has presented as one of the key functional impairments among those with ADHD. Indeed, institutional records of critical incidents have shown a strong association between ADHD symptoms and aggressive behaviours in males with and without mental disorders detained in forensic settings [9],[10]. In a study conducted in a Scottish prison, compared with their Non-ADHD peers, participants with ADHD symptoms were involved in a significantly higher total number of critical incidents, including incidents of verbal aggression and physical aggression with significantly more severe outcomes [10]. A study of males with personality disorder detained in secure hospital services found similar results; those reporting ADHD symptoms had a significantly greater frequency of critical incidents, in particular those involving verbal aggression and damage to property [9].

Retz and Rösler[11] propose that the relationship between ADHD symptoms, conduct disorder and aggression is associated with impulsive aggression as a reaction to a situation, rather than with premeditated proactive aggression. This is supported by the findings of Dowson and Blackwell[12] who found that 40% of male outpatients endorsed impulsive externally directed aggression and 47% reported impulsive internally directed aggression (i.e. aggression directed towards the self, including self-harming and suicidal ideations or behaviours). Inattention, and in particular hyperactivity and impulsivity were all predictors of externally directed aggression.

Impulsivity has been associated with self-harming behaviours. Research has identified strong associations between impulsivity, aggression and suicidal behaviour within psychiatric populations [13],[14]. A positive correlation has been identified between impulsivity and suicidal behaviour [15], and a higher risk of suicide has been reported in individuals with adult ADHD [16],[17]. The most robust evidence of an association between ADHD and suicide comes from a meta-analysis by Impey and Heun[18], who reported higher rates of suicidal ideation and suicide attempts within ADHD samples compared with controls. However, much of the current literature has focused on the association between ADHD and behavioural disturbance in male offender populations. Research has identified that women differ from men in ADHD symptom profile and this difference has been partially attributed to hormonal factors [19]. There has been little research into the type of behavioural incidents that may arise in female offenders with ADHD.

This pilot study therefore aimed to further our understanding of ADHD within a cohort of female mentally disordered offenders by ascertaining estimated rates of ADHD and associated behavioural problems presenting in this population. The study utilised ADHD screening tools to determine childhood and current adult ADHD symptomology. Behavioural disturbance was identified from a review of patients' clinical notes.

Consistent with the literature, it was hypothesised that: (H1) rates of estimated ADHD would be considerably higher than that reported in the general population for females; (H2) that female mentally disordered offenders with ADHD symptoms would have greater behavioural impairments (i.e., aggressive and self-harming behaviours) compared with their Non-ADHD peers; and (H3) that there would be a positive correlation between behavioural disturbance and ADHD symptoms and, in particular, hyperactive/impulsive symptoms.

## 2. Materials and Methods

### 2.1 Participants

The study took place over a 14-month period at an adult medium secure hospital. Fifty females between 18 and 52 years of age, (*M* = 30.85, *SD* = 9.81) who were detained under the UK Mental Health Act participated in the study. Exclusion criteria included patients who were too mentally unstable to participate at the time of the assessment, or who had severe cognitive deficits and/or who lacked capacity to consent to participate in the study. A cohort of eighty patients was available for participation. In two cases, the nursing staff and clinical team advised that the patients were in acute psychological distress and were therefore too mentally unwell to approach. Twenty eight patients did not give consent.

The primary diagnoses of the 50 patients who agreed to participate were as follows: 30% (N = 15) schizophrenia, 26% (N = 13) emotionally unstable personality disorder, 16% (N = 8) schizoaffective disorder, 10% (N = 5) bipolar disorder, 10% (N = 5) post-traumatic stress disorder, two patients had Asperger's Syndrome, one had depression and one had dissocial personality disorder.

Participants were prescribed a range of medication, including anti-psychotics, anti-depressants, mood stabilisers, benzodiazepines and ADHD stimulant medications. A total of fourteen patients (28% of the total sample) were being treated with stimulant medication during the assessment period.

### 2.2 Measures

The self-rated Barkley ADHD Scales [20] measure the retrospective report of childhood symptoms and the current report of adult symptoms. The scales include 18 items (9 in each domain of inattention and hyperactivity/impulsivity), which are retrospectively phrased for childhood symptoms. The responses for each item are scored on a four point rating scale (0 = ‘Never or Rarely’, 1 = Sometimes, 2 = Often and 3 = ‘Very Often’). For the purposes of classification, scores of 2 or 3 indicate that a symptom is present. Consistent with DSM-5 [1] patients with six or more symptoms in either or both domains in childhood AND five or more symptoms in either or both domains in adulthood were classified as positive (ADHD group). Using this methodology, classifications of subtype based on current symptoms were made for the domains of predominantly hyperactive/impulsive subtype (i.e., those meeting the hyperactive/impulsive but not the inattentive domain)*,* predominantly inattentive subtype (i.e., those meeting the inattentive but not the hyperactive/impulsive domain), or combined subtype (i.e., those meeting criteria for both domains). In addition, all ratings were summed to provide a total Barkley score for each participant.

Demographic and behavioural data were obtained from a review of clinical files documenting primary diagnosis, age, length of stay (in years) at the hospital, and the frequency and severity of aggressive and self-harming behaviours recorded over the 12 weeks prior to participation in the study. Behavioural disturbance was categorised using the previous methodology of Young and colleagues [9], which recorded the frequency of verbal aggression, physical aggression, damage to property and arson. In addition, the frequency of deliberate self-harming behaviour was recorded as: verbal intent to self-harm or expression of suicidal ideation, superficial injury, cutting, ligatures, and insertion of foreign objects. The severity of physical aggression and deliberate self-harm was rated on a 1–4 scale as follows:

1. A verbal threat without actualisation.
2. Harm to self or others not requiring response team/medical attention.
3. Harm to self or others requiring response team/medical attention.
4. Harm to self or others requiring hospitalisation.

Composite scores were calculated for aggressive behaviour and self-harming behaviour by adding the total frequency of incidents and the severity rating for these incidents. A total composite score of behavioural disturbance, combining both aggressive and self-harming behaviour, was also calculated for each patient. Thus, seven main measures were calculated: Frequency of Aggression, Severity of Aggression, Composite Score of Aggression, Frequency of Self-Harm, Severity of Self-Harm, Composite Score of Self-Harm, and Total Composite Score.

### 2.3 Procedure

The study was approved by the South East London Research Ethics Committee and by the Research and Development Office, King's College London, (08/HO807/17 and R&D2008/007 respectively). Prior to their recruitment, patients were given an information sheet regarding the nature and purpose of the study and were given the opportunity to ask questions about the research. After giving informed written consent, patients completed the ADHD screens with the researcher. The demographic and behavioural data were then obtained from a review of their clinical records.

### 2.4 Statistical analysis

All statistical analyses were performed using SPSS for Windows, Version 20. Descriptive statistics were used to characterise the sample. Mann-Whitney U tests were conducted to compare differences in behavioural disturbance between the ADHD group and the Non-ADHD Group. Spearman's Rank Order Correlation analysis was used to examine the relationship between ADHD subtypes and behavioural disturbance. One tailed tests with the significant level set at 0.05 were used to test hypotheses.

## 3. Results

### 3.1 Estimated prevalence of adult ADHD

Just under one-third (28%; N = 14) of the sample met the screening criteria for adult ADHD, based on childhood and current symptoms. Of these, 43% (N = 6) were classified as hyperactive/impulsive subtype, 43% (N = 6) as combined subtype and; the remaining 14% (N = 2) of patients were of the inattentive subtype.

The ADHD Group were significantly younger (*M* = 24.59, *SD* = 5.11) than the Non-ADHD Group (*M* = 33.29, *SD* = 10.17; *t*(44.59) = 4.00, *p* < 0.001). There were no significant differences between the groups for length of stay (*M* = 1.37, *SD* = 1.26 and *M* = 1.72, *SD* = 2.02 respectively, *t*(47) = 0 .60, NS). Data was missing for one case from the Non-ADHD group.

The Non-ADHD Group was comprised of 36 patients, 52.8% (N = 19) with primary diagnoses of severe mental illness (SMI; schizophrenia, schizoaffective disorder and bipolar disorder) and 47.2% (N = 17) had Non-SMI primary diagnoses. Around two thirds (64.3%; N = 9) of the ADHD Group was comprised of patients with primary diagnoses of SMI and the remaining 35.7% (N = 5) of patients had Non-SMI primary diagnoses.

### 3.2 Behavioural disturbance

Mann-Whitney *U* tests were conducted to assess for differences in the total number and severity of incidents between the ADHD and Non-ADHD Groups (see Table 1). Participants with ADHD had higher scores in all measures of behavioural disturbance; however these differences were not statistically significant.

**Table 1.** Incidents and Severity of Aggressive and Self-Harming Behaviour.

	ADHD Group(N = 14)	Non-ADHD Group(N = 36)	
Behavioural Disturbance	Mean (*SD*)	Mean (*SD*)	*𝓏*
Frequency of Aggression	4.93 (7.25)	2.19 (2.87)	−0.428
Severity of Aggression	6.36 (10.88)	2.42 (3.52)	−0.510
Composite Aggression	11.29 (17.60)	4.61 (6.12)	−0.416
Frequency of Self-Harm	3.57 (5.83)	1.50 (2.31)	−0.738
Severity of Self-Harm	7.57 (13.00)	2.94 (4.89)	−0.888
Composite Self-Harm	11.14 (18.77)	4.42 (7.17)	−0.871
Total Composite Score	22.43 (31.08)	9.03 (8.81)	−0.999

To control for the effect of age, age-adjusted residuals were entered into Spearman's correlation as the data were not normally distributed (Table 2). The total ADHD symptom score, obtained from the Barkley scale that screened for ‘current’ symptoms, significantly and positively correlated with all of the behavioural disturbance measures except for the Frequency of Aggression score. There was a significant positive correlation between the hyperactive/impulsive symptoms and all behavioural disturbance measures. Inattentive symptoms did not significantly correlate with these measures.

**Table 2.** Spearman's Rank Order Correlation for adult ADHD Symptoms and Behavioural Disturbance.

	Inattentive symptom score (N = 50)	Hyperactive/Impulsive symptom score (N = 50)	Total Barkley score (N = 50)
Behavioural Disturbance	Spearman *r_Ѕ_*	Spearman *r_Ѕ_*	Spearman *r_Ѕ_*
Frequency of Aggression	0.117	0.255^*^	0.234
Severity of Aggression	0.168	0.244^*^	0.263^*^
Composite Aggression	0.173	0.250^*^	0.244^*^
Frequency of Self-Harm	0.220	0.337^**^	0.333^**^
Severity of Self-Harm	0.193	0.304^*^	0.337^**^
Composite Self-Harm	0.213	0.335^**^	0.346^**^
Total Composite Score	0.232	0.363^**^	0.381^**^

*p < 0.05, **p < 0.01 (*one-tailed*)

## 4. Discussion

This study set out to investigate the estimated rate of ADHD and associated behavioural disturbance within a mentally disordered female population. As hypothesised, the screening rate was significantly higher than that reported in the general population. Almost one-third (28%) screened positively for ADHD, most of whom met screening criteria for either hyperactive/impulsive subtype (43%) or combined subtype (43%), highlighting the considerable presence of hyperactivity and impulsivity within this population. However, screening questionnaires alone may lack specificity for the diagnosis, with high rates of false positives having been identified in previous research [21]. Due to the complexity of the current patient group, higher rates of false positives could be expected within this sample. The sensitivity of rating scales for predicting a diagnosis (obtained from a comprehensive clinical assessment) is unknown, and it is possible that false negatives may also have been present. Future research should include a clinical assessment, such as the Diagnostic Interview for ADHD in Adults [22] to obtain a definitive categorisation of the disorder.

The second hypothesis was not supported as levels of behavioural disturbance did not significantly differ between groups. However, although no significant differences were found between the mean scores, the ADHD group obtained higher ratings for frequency and severity of aggressive and self-harming behaviours than the non-ADHD group and it is possible that an effect may have been found with a larger sample. The ADHD group had considerably greater variation in their behaviour scores and were significantly younger. Future research should employ a larger sample size and/or match groups by age. When controlling for age, hyperactive/impulsive and total Barkley score were significantly correlated with all measures of behavioural disturbance, with the exception of the Frequency of Aggression, partly supporting the third hypothesis. Inattentive symptoms did not correlate significantly with the measures of behavioural disturbance, although all correlations were in the positive direction. These findings suggest that ADHD symptom scores are associated with increased aggressive behaviour, and that this effect appears to be due to hyperactive/impulsive symptoms rather than inattention symptoms alone. Further to this, and a novel finding, the ADHD symptoms were more strongly correlated with self-harm than outward aggression (medium versus low effect size). This differs from previous research into male prisoners, where the observation of self-harm is very rare and is not related to ADHD symptoms [10]. The underlying mechanism that drives the relationship between ADHD symptoms and self-harm in the current study is unknown, but likely involves emotional instability in addition to impulsivity, since emotional dysregulation is known to be a common associated feature of ADHD in adults, and is related more strongly to the hyperactive-impulsive symptoms of the disorder [23].

This study highlights the potential impact of ADHD symptoms among female offenders with severe mental health disorders, a population that has hardly been studied. Yet there are several limitations that should be addressed in future research. The current pilot is limited by its small sample size; recruitment was hampered by the high refusal rate (35%) and this should be factored in when planning future studies in a similar population. As a result, the study may have been vulnerable to selection bias, limiting generalisability of the findings. Whilst screening tools may have utility to identify those who require a more comprehensive assessment, they have the limitations acknowledged above and those measures that require retrospective recall of childhood symptoms may be susceptible to memory bias [24]. This pilot is a naturalistic study and the participants were prescribed a range of medication; fourteen patients (28% of the total sample) were treated with stimulant medication and half of these were in the Non-ADHD patient group, reflecting clinical judgments about treatment based on long term assessment of the patients within the forensic setting. Since stimulants are an effective treatment for ADHD this is the likely explanation for the low rate of ADHD in our study among the patients with a hospital diagnosis of ADHD. Stimulants are expected to reduce current levels of ADHD symptoms to subthreshold levels. The fact that 50% still have ADHD is within the expected range of partial and non-responders identified in previous clinical trials. A strength of the current study is that behavioural disturbance data were obtained from staff reported behaviours recorded in the clinical records over a twelve week period.

## 5. Conclusion

This pilot study of a cohort of patients detained at a medium secure service has contributed to the knowledge base about the rate and functional problems of female offenders with ADHD. Future research should replicate the study using a larger sample and explore the effect of treatment (pharmacological and psychological) on the reduction of ADHD symptoms, behavioural disturbance and quality of life. Treatment that combines both pharmacological and psychological approaches, in particular, is likely to be necessary to improve the functional long-term outcomes of individuals with complex presentations. In turn this has the potential to reduce the length of stay in costly secure mental health hospitals.

## References

[1] American Psychiatric Association (2013). Diagnostic and statistical manual of mental disorders,.

[2] Simon V, Czobor P, Balint S (2007). Prevalence and correlates of adult attention-deficit hyperactivity disorder: meta analysis. Brit J Psychiat.

[3] De Zwaan M, Gruss B, Müller A (2012). The estimated prevalence and correlates of adult ADHD in a German community sample. Eur Arch Psy Clin N.

[4] Faraone SV, Biederman J (2005). What is the prevalence of adult ADHD? Results of a population screen of 966 adults. J Atten Disord.

[5] Young S, Adamou M, Bolea B (2011). The identification and management of ADHD offenders within the criminal justice system: a consensus statement from the UK Adult ADHD Network and criminal justice agencies. BMC Psychiatry.

[6] Cahill BS, Coolidge FL, Segal DL (2012). Prevalence of ADHD and Its Subtypes in Male and Female Adult Prison Inmates. Behav Sci Law.

[7] Konstenius M, Larsson H, Lundholm L (2012). An Epidemiological Study of ADHD, Substance Use, and Comorbid Problems in Incarcerated Women in Sweden. J Atten Disorddoi.

[8] Rösler M, Retz W, Yaqoobi K (2009). Attention deficit/hyperactivity disorder in female offenders: prevalence, psychiatric comorbidity and psychosocial implications. Eur Arch Psy Clin N.

[9] Young S, Gudjonsson G, Ball S (2003). Journal of Forensic Psychiatry & Attention Deficit Hyperactivity Disorder (ADHD) in personality disordered offenders and the association with disruptive behavioural problems. J Forensic Psychiatry.

[10] Young S, Gudjonsson G, Wells J (2009). Attention deficit hyperactivity disorder and critical incidents in a Scottish prison population. Personality Individual Dif.

[11] Retz W, Rösler M (2007). Gewalttätiges Verhalten bei Straftätern mit ADHS: Assoziation mit reaktiver, nicht aber proaktiver Gewalt. Der Nervenarzt.

[12] Dowson JH, Blackwell AD (2010). Impulsive aggression in adults with attention-deficit/hyperactivity disorder. Acta Psychiat Scand.

[13] Conner KR, Duberstein PR, Conwell Y (2001). Psychological vulnerability to completed suicide: a review of empirical studies. Suicide Life Threat Behav.

[14] Gvion Y, Apter A (2011). Aggression, impulsivity, and suicide behavior: a review of the literature. Arch Suicide Res.

[15] Apter A, Plutchik R, van Praag HM (1993). Anxiety, impulsivity, and depressed mood in relation to suicidal and violent behavior. A Acta Psychiat Scand.

[16] Goldston DB, Daniel SS, Erkanli A (2009). Psychiatric diagnoses as contemporaneous risk factors for suicide attempts among adolescents and young adults: developmental changes. J Consult Clin Psych.

[17] Westmoreland P, Gunter T, Loveless P (2010). Attention deficit hyperactivity disorder in men and women newly committed to prison: clinical characteristics, psychiatric comorbidity, and quality of life. Inter J Offen.

[18] Impey M, Heun R (2012). Completed suicide, ideation and attempt in attention deficit hyperactivity disorder. Acta Psychiat Scand.

[19] Nussbaum NL (2012). ADHD and female specific concerns: A review of the literature and clinical implications. J Attent Disord.

[20] Barkley RA, Fischer M, Smallish L (2002). The persistence of attention-deficit/hyperactivity disorder into young adulthood as a function of reporting source and definition of disorder. J Abnorm Psychol.

[21] McCann BS, Roy-Byrne P (2004). Screening and diagnostic utility of self-report attention defecit hyperactivity disorder scales in adults. Compr Psychiat.

[22] Kooij JJS, Francken MH (2010). DIVA 2.0. Diagnostic Interview Voor ADHD in Adults bij volwassenen [DIVA 2 0 Diagnostic Interview ADHD in Adults].

[23] Asherson P, Skirrow C, McLoughlin G (2010). The co-variation of mood variability with ADHD symptoms. Eur Psychiat.

[24] Mannuzza S, Klein RG, Klein DF (2002). Accuracy of Adult Recall of Childhood Attention Deficit Hyperactivity Disorder. Am J Psychiat.

